# Statistics

**Published:** 1900-04-21

**Authors:** 


"STATISTICS."

The misleading nature of many of the statistics put forward by those who make opposition to scientific medicine their work in life, is well shown up in a letter by Dr. Richard T. Hewlett, which was published in the *Lancet* last week (April 14th, 1900). It appears from this that the Hon. Stephen Coleridge has widely published the statement that, according to the figures in the Registrar General's reports, the annual deaths from diphtheria had increased from 3,992 in the year 1882, to 7,654 in 1897, and that the death-rate from diphtheria per 1,000,000 living had increased in the same time from 152 to 246, his object being, of course, to discredit the antitoxin treatment. Now Dr. Hewlett says, as everyone ought to know who deals with such a subject, that diphtheria and croup must be considered together, and, as showing how well recognised this is, he gives the following extract from the Report of the Registrar General: "It is safe to assume that the greater part of the deaths referred in the certificates to croup, as well as some of those referred to laryngitis and to ill-defined sore throat, were really due to diphtheria." Indeed, in the very Report of the Registrar General from which Mr. Coleridge takes his figures, the deaths from diphtheria and croup are placed together in one table—one which shows that, taken together as they are there given, so far from the death-rate from these diseases increasing it has actually diminished during the years mentioned. We do not ourselves believe that these figures, whichever way they are taken, have any real bearing upon the question of the antitoxin treatment, but what is quite clear is that if such figures are to be accepted as showing anything, so far from supporting Mr. Coleridge's views, they do the very reverse.

